# Chemokines are secreted by monocytes following OK-432 (lyophilized *Streptococcus pyogenes*) stimulation

**DOI:** 10.1186/1471-2172-10-6

**Published:** 2009-01-28

**Authors:** Carla Olsnes, Helen Stavang, Karl Brokstad, Jan Olofsson, Hans J Aarstad

**Affiliations:** 1 Department of Surgical Sciences, Faculty of Medicine, University of Bergen, Jonas Lies vei 66, 5021, Bergen, Norway; 2 Department of Head and Neck Surgery, Haukeland University Hospital, 5021, Bergen, Norway; 3 Broegelmann Research Laboratory, Gades Institute, University of Bergen, 5021, Bergen, Norway

## Abstract

**Background:**

OK-432, penicillin-killed *Streptococcus pyogenes*, is used in treating lymphangiomas and carcinomas. We have studied *in vitro *the role of mononuclear phagocytes (MNPs), including purified monocytes (MOs), in the immune response to OK-432. MIP-1α/β and MCP-1 secretions were assessed in whole blood (WB), peripheral blood mononuclear cells (PBMCs) and purified MOs, after *in vitro *stimulation with OK-432 with or without adherence for 24 hours.

**Results:**

OK-432 stimulated MNPs to secrete MCP-1 and MIP-1α/β in healthy individuals and in head and neck squamous cell carcinoma (HNSCC) patients, except for OK-432 stimulation of WB giving a minimal MIP-1α/β response. Upon culture on low-attachment wells, a spontaneous chemokine secretion was observed, with an unchanged secretion following OK-432 stimulation. Inhibition of Syk kinase and/or PI-3 kinase did not significantly change the chemokine response to OK-432, except for MIP-1α production being increased upon Syk inhibitor addition and an increased MCP-1 response upon addition of both inhibitors. Adhesion may possibly involve β_1 _and/or β_3 _integrins, not β_2_, whereas β_1–3 _integrins may act as co-stimulatory receptors for OK-432. Based on direct blockage of CD36 or CD18 by antibodies, MCP-1 production may be mediated by CD18 while MIP-1β and MCP-1 production may occur upon binding to CD36.

**Conclusion:**

Adherent human MOs produce MCP-1 and MIP-1α/β upon stimulation with OK-432. CD36 modulates MIP-1β and MCP-1 response. Thus, to some extent OK-432 acts as a substance whereby only MOs adhered to surfaces secrete MCP-1 and MIP-1α/β, in part explaining why OK-432 is suited as a biological response modifying drug.

## Background

The innate immune system detects and eliminates invading foreign material through non-specific defense mechanisms elicited by, e.g., mononuclear phagocytes (MNP). MNPs originate as monoblasts in the bone marrow, reside as monocytes (MOs) in blood and become, e.g., tissue macrophages (Mϕs) or dendritic cells (DCs) upon extravasation into tissues. The innate immune system is presumably involved when biological response modifiers (BRMs) are utilized in the treatment of diseases such as cancer [1,2] and lymphangiomas [3]. Killed bacterial toxins [4] along with bacillus Calmette-Guerin (BCG) [5], β-glucan [6], interferons [7] and monoclonal antibodies [8] are examples of BRMs used in cancer treatment.

Japan has a long standing tradition in using penicillin-killed lyophilized *Streptococcus pyogenes*, denominated OK-432 or picibanil, as a biological response modifier (BRM) for treatment of cancer [1,2]. Sakamoto *et al. *[1] published in 2001 a meta-analysis showing a 20% 5-year survival improvement with immunochemotherapy, compared to chemotherapy alone, following OK-432 treatment in patients diagnosed with non-small-cell lung cancer. This meta-analysis was based on 1,520 patients enrolled in 11 randomized clinical phase III trials. Furthermore, Oba et al [2] published in 2007 a meta-analysis including 8009 gastric carcinoma patients from 8 randomized clinical phase III trials and concluded that compared to control conditions, addition of OK-432 treatment increased survival. There are also reports suggesting that patients with other cancers, such as head and neck squamous cell carcinoma (HNSCC) [9], may benefit from OK-432 treatment. OK-432 has also been used as a maturation factor for DCs cells as part of vaccination therapy of cancer patients [10].

Lymphangiomas are benign neoplasias of lymphatic origin, often congenital, that may extend around vital structures [11]. Surgical removal of lymphangiomas has been the standard treatment, but may be technically difficult. Injection of OK-432 into cystic lymphangioma lesions may lead to shrinkage and subsequent cure as first reported by Ogita [3]. Today, injection with OK-432 is established as a major treatment option for lymphangiomas [11], and is an important example of the use of BRMs in medicine.

OK-432 presumably exerts its effect by activating the immune system to secrete toxic substances, which in turn eliminate tumor cells [12]. It is, however, relatively little known about the mechanisms of action of OK-432. The principal cells responding to the drug, the engaged receptors or signal transduction pathways, are to a surprisingly large extent unknown. This should be an important area of study. The efficiency of OK-432 treatment needs to be improved in order to make OK-432 a better cancer treatment drug. Additional drugs, which could improve OK-432's response, as well as studying to what extent other (cancer) drugs potentially interfere with the receptors and signal transduction pathways driving the OK-432 response, should therefore be determined as to optimize and improve treatment.

Furthermore,*S. pyogenes *is an important pathogen causing human disease. The diseases caused by *S. pyogenes *range from tonsillitis, impetigo, necrotizing fasciitis, and scarlet fever to lethal toxic shock syndrome [13]. Thus, studying the interactions of OK-432 with the immune system may shed light into the biology of these diseases as well.

One major MNP function is secretion of chemokines, such as monocyte chemoattractant protein (MCP) -1 and macrophage inflammatory protein (MIP) -1α/β [14]. Chemokines have been shown to be secreted in response to OK-432 stimulation [15] and have the main effect of attracting new leucocytes to the site from where they are secreted [16]. It should be of interest, to determine the modality of chemokine secretion from MOs and Mϕs upon OK-432 stimulation. We have therefore found it pertinent to address this issue in the present investigation.

Priming provided by adherence, presumably associated with MNP differentiation from MO to Mϕ, contributes to interleukin secretion following OK-432 MNP stimulation [17]. Therefore, it is of interest to study to what extent the MNP chemokine response to OK-432 depends on adherence. Molecularly, we have found it relevant to investigate the role of β-integrin receptors, as these receptors participate during differentiation of MNP from MO to Mϕ. Furthermore, these receptors may be functionally blocked by addition of piceatannol (Syk kinase inhibitor) and/or LY294002 (phosphoinositide (PI)-3 kinase inhibitor) [18-22]. OK-432 may stimulate TLR receptors. In addition to OK-432, we have studied chemokine secretion upon stimulation with the known TLR2 agonist LTA and TLR4 agonist LPS. Moreover, we explored the role of the TLR2 co-receptor CD36 [23] in OK-432 stimulation.

We have determined that *in vitro *purified adherent MNPs may secrete chemokines MCP-1, MIP-1α and MIP-1β following OK-432 stimulation. MOs deprived of adherence did not respond to OK-432 stimulation as assessed by chemokine secretion. Inhibition of Syk and PI-3 kinase did not block the stimulatory effect of OK-432. MIP-1α/β production in WB and MOs was higher upon stimulation with LTA or LPS while OK-432 stimulation gave a higher MCP-1 secretion in WB and MOs in comparison with LTA or LPS. CD36, and to some extent CD18, participate in the OK-432-modulated MNP chemokine response.

## Results

### In vitro chemokine production in unstimulated cultures

**Figure 1:**
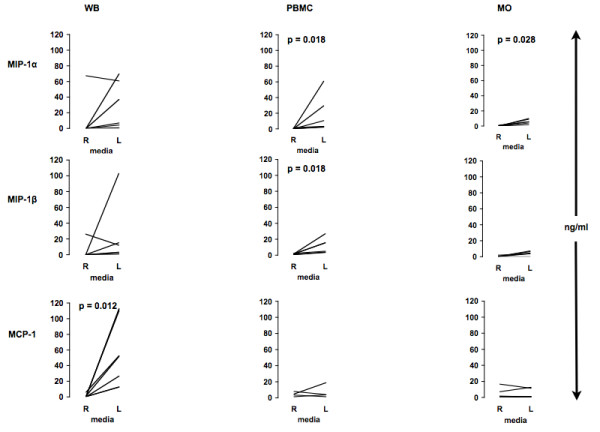
**In vitro chemokine production in unstimulated WB, PBMCs or MOs**. Human WB, PBMCs or MOs were plated in parallel on regular (R) or low attachment (L) plates and left unstimulated for 24 h (A). Supernatants were then collected and analyzed using Multiplex (MIP) or ELISA (MCP-1). WB (n = 8); PBMC (MIP-1α/β: n = 7; MCP-1: n = 4); MO (n = 6).

Upon adherence, only MCP-1 was to some extent secreted (Fig. 1). Upon low adherence conditions, MCP-1 and MIP-1α/β were secreted at all studied conditions (Fig. 1).

### Chemokine production following OK-432 stimulation

**Figure 2:**
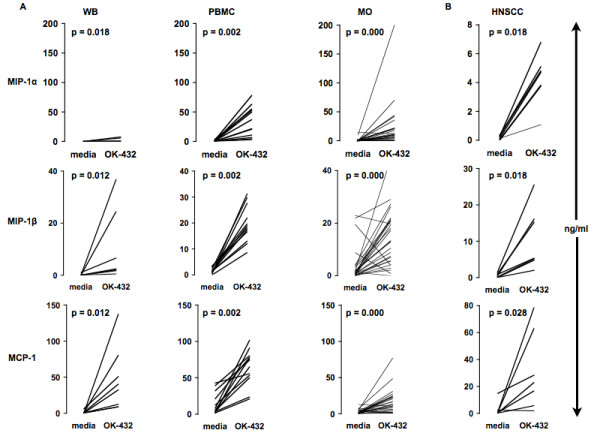
**OK-432 stimulates release of chemokines in WB, PBMCs, and isolated human MOs from healthy donors and HNSCC patients**. Human WB, PBMCs or MOs were stimulated with media or OK-432 (0.01 KE/ml) for 24 h (A). MOs isolated from four HNSCC patients were stimulated for 24 h with media or OK-432 (0.01 KE/ml) (B). Supernatants were then collected and analyzed using Multiplex (MIP) or ELISA (MCP-1). A: WB (n = 8); PBMC (n = 12); MO (n = 27); B: HNSCC (n = 4).

**Figure 3:**
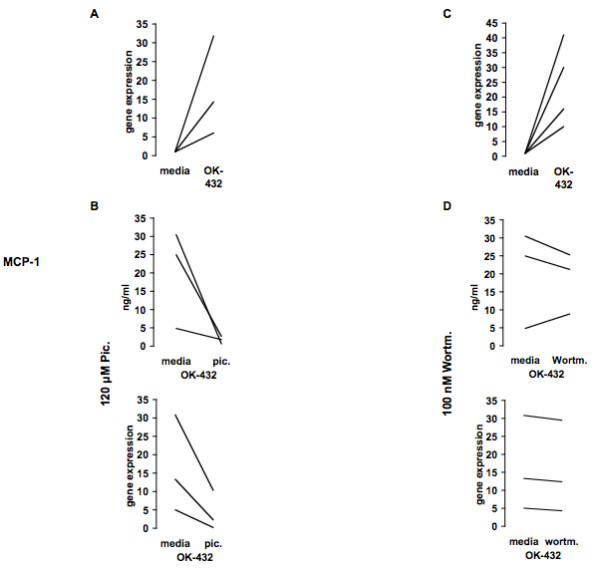
**OK-432 augments MCP-1 gene expression in MOs from healthy donors and HNSCC patients and correlates with MCP-1 protein levels upon inhibition with piceatannol and wortmannin**. MCP-1 gene expression was assessed in MOs stimulated with OK-432 for 24 h from three randomly selected healthy donors (A) or two HNSCC patients on two separate days (C). In separate experiments, MOs, stimulated with piceatannol (120 μM) (B) or wortmannin (100 nM) (D) for 30 min followed by stimulation with OK-432 for an additional 24 h, were analyzed by ELISA or real-time PCR with respect to MCP-1 production and gene expression, respectively. A, B, and D: n = 3; C: n = 2.

At all tested regular growth conditions, OK-432 stimulated the release of MIP-1α/β, and MCP-1 (all p < 0.05) (Fig. 2). We determined a similar secretion for MCP-1 and MIP-1α/β upon stimulating PBMCs versus MOs (Fig. 2). Stimulated WB gave a lower MIP-1α secretion compared to PBMCs or MOs (Fig. 2). The effect of OK-432 was also tested with MOs from HNSCC patients giving the same chemokine response profile as healthy controls (Fig. 2). With respect to MCP-1, close correlation between protein levels and mRNA levels was observed in three healthy donors (Fig. 3A) as well as two HNSCC patients, tested on two separate days (Fig. 3C) suggesting a pre-transcriptional regulation upon *in vitro *OK-432 stimulation of MOs.

### Chemokine production following OK-432 versus LTA or LPS stimulation

**Figure 4:**
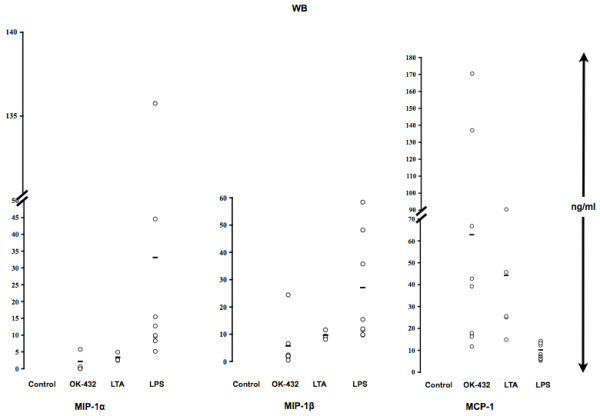
**Chemokine production follows OK-432, LTA or LPS in vitro stimulation of WB**. Human WB was stimulated in parallel with OK-432 (0.01 KE/ml), LTA (0.5 μg/ml), LPS (1 μg/ml), or left unstimulated for 24 h. Supernatants were then collected and analyzed using Multiplex (MIP) or ELISA (MCP-1). Control, OK-432, LPS: n = 8; LTA: n = 4.

**Figure 5:**
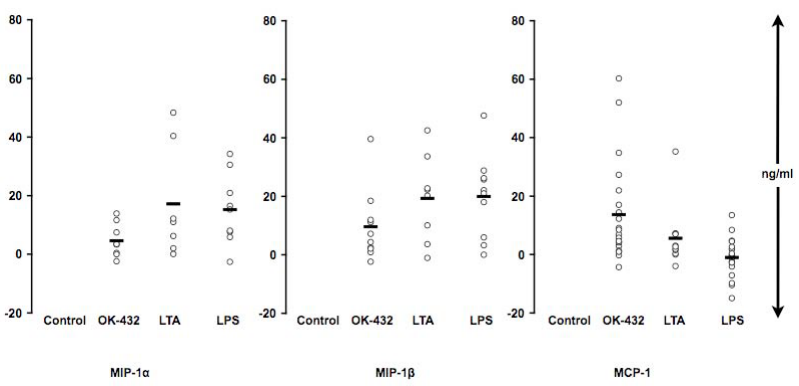
**OK-432, LTA or LPS in vitro stimulation of MOs**. MOs were stimulated in parallel with OK-432 (0.01 KE/ml), LTA (0.5 μg/ml), LPS (1 μg/ml), or left unstimulated for 24 h. Supernatants were then collected and analyzed using Multiplex (MIP) or ELISA (MCP-1). MIP-1α (Control, OK-432, LPS: n = 9; LTA: n = 7).MIP-1β (Control, OK-432, LPS: n = 10; LTA: n = 8); MCP-1 (Control, OK-432: n = 19; LTA: n = 9; LPS: n = 16).

Tested were WB (Fig. 4) and MOs (Fig. 5) stimulated in parallel with OK-432 or LTA (TLR2 ligand) or LPS (TLR4 ligand). In WB stimulated with OK-432 or LTA, the response was primarily seen with MCP-1, and only to some extent with MIP-1α/β, whereas the LPS-stimulated response was determined by MIP-1α/β secretion. In purified MOs, OK-432 and LTA stimulated secretion of MCP-1. LPS inhibited MCP-1 production. Thus, it appears that OK-432 stimulation is more akin to LTA than to LPS stimulation.

### Chemokine production following OK-432 stimulation of WB, PBMCs and MOs cultured on regular or low-attachment culture wells

**Figure 6:**
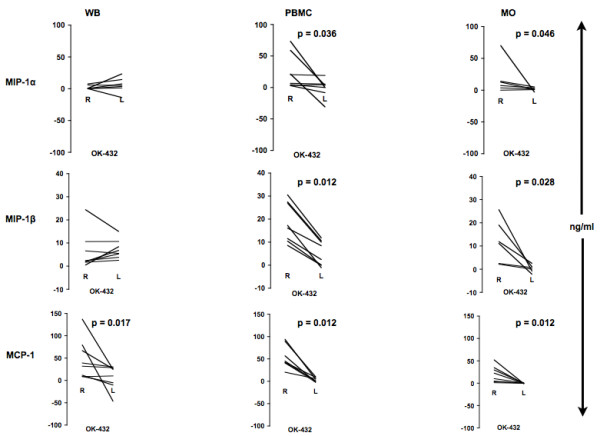
**OK-432 stimulation of adherent or suspended WB, PBMCs, and MOs**. WB, PBMCs or MOs were stimulated with OK-432 (0.01 KE/ml) or left unstimulated on regular (R) or low-attachment (L) plates for 24 h. Supernatants were analyzed using Multiplex (MIP) or ELISA (MCP-1). WB: n = 8; PBMC: n = 8; MO: n = 6.

Chemokine secretion was compared in WB and MNPs cultured on regular (R) or low-attachment (L) wells (Fig. 6). In WB, the MCP-1 response was reduced by 90% at the L compared to the R condition (48 ± 4 vs 7 ± 27 ng/ml; p < 0.02). In PBMCs, MIP-1α response was abolished at the L condition 24 ± 9.7 vs -1 ± 5.5 ng/ml; p < 0.04). Furthermore, MIP-1β response was reduced by 74% (19 ± 3 vs 5 ± 2 ng/ml; p < 0.02), and the MCP-1 response by 94% (54 ± 9 vs 3.1 ± 1.7 ng/ml; p < 0.02). In MOs, MCP-1 (19.8 ± 6.4 vs -0.2 ± 0.2 ng/ml; p < 0.02) response was completely blocked, while MIP-1α was reduced by 92% 17.9 ± 10.7 vs 1.5 ± 1.1 ng/ml; p < 0.05) and MIP-1β by 98% 12.1 ± 3.8 vs 0.2 ± 0.6 ng/ml; p < 0.03). Thus, it appears that chemokine responses following OK-432-stimulation are almost absent if the cells are minimally allowed to adhere to a surface.

### Chemokine production following OK-432 stimulation of PBMC, MO-depleted PBMCs or MOs

**Figure 7:**
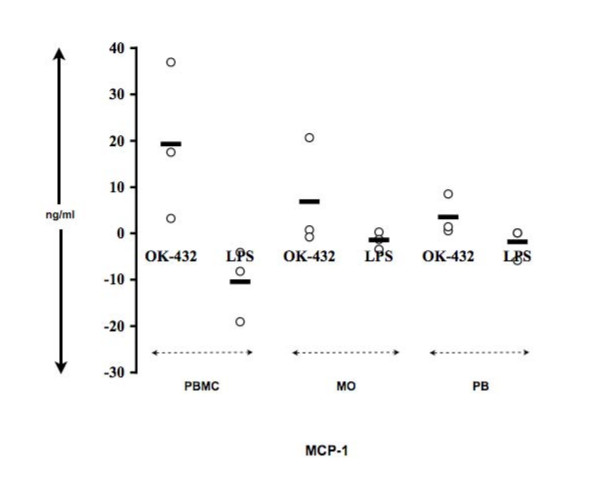
**MO-depleted PBMCs secrete less chemokines than PBMCs upon OK-432 stimulation**. PBMCs, MOs, or PBs (MO-depleted PBMCs) were left unstimulated or stimulated in parallel with OK-432 (0.01 KE/ml) or LPS (1 μg/ml) for 24 h. Supernatants were analyzed using Multiplex (MIP) or ELISA (MCP-1). n = 2.

Chemokine secretion was determined from PBMCs, MOs separated by adherence and MO-depleted PBMCs (Fig. 7). MO-depleted PBMCs (PB) and MOs secreted 18% and 36% of the PBMC-derived MCP-1, respectively (Figure 7). Thus, MOs are the major source for MCP-1 production. In contrast, PBMCs stimulated with LPS gave a negative MCP-1 production since stimulation with LPS gives lower MCP-1 secretions compared to media controls.

### Stimulation of MOs with OK-432 is independent of Syk and PI-3 kinase

**Figure 8:**
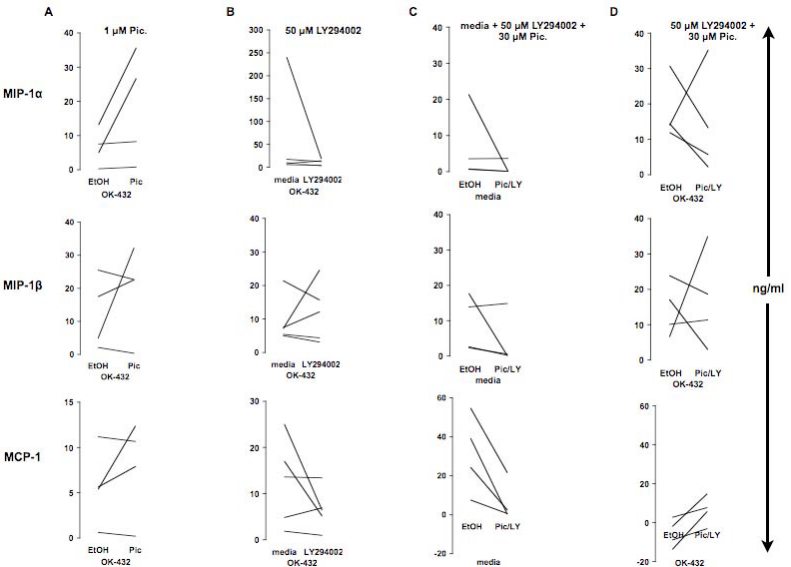
**Syk and PI-3 kinase-independent signaling for OK-432 stimulation of MOs**. Piceatannol (1 μM) or diluent control was added to MO cultures for 30 min prior to addition of OK-432 for 24 h (A). LY294002 (50 μM) was added to MOs for 30 min prior to addition of OK-432 for 24 h (B). In separate experiments, a combination of LY294002 (50 μM) and piceatannol (30 μM) was added to whole blood and throughout the monocyte isolation procedure (C) and followed by stimulation with OK-432 or left unstimulated for 24 h (D). Supernatants were analyzed using Multiplex (MIP) or ELISA (MCP-1). A: n = 4; B: n = 5; C: n = 4; D: n = 4.

**Figure 9:**
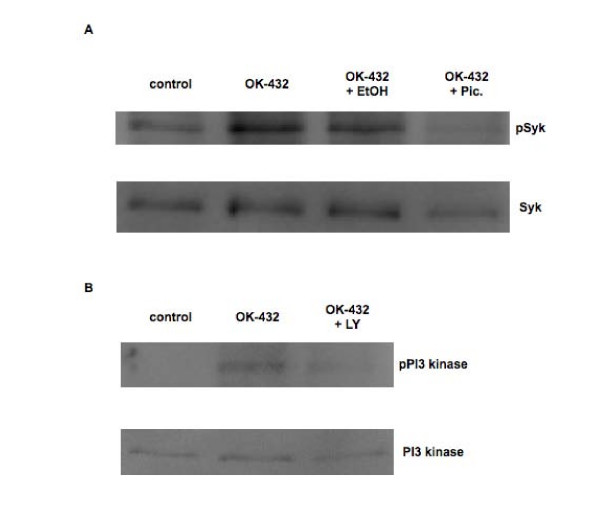
**OK-432 promotes Syk or PI-3 kinase phosphorylation in MOs**. MOs, isolated by the monocyte negative isolation kit, were incubated either with piceatannol, Pic. (30 μM) or diluent control (A) or LY294002, LY (50 μM) (B) and either left unstimulated or further stimulated with OK-432 for 15 min. Blots shown are representative of two separate experiments performed with pooled MOs obtained from two different blood donors.

In order to determine whether Syk kinase activation was necessary for the observed chemokine production in OK-432-stimulated MOs, the Syk kinase inhibitor piceatannol (120 μM, (Fig. 3B), 30 μM (not shown), 1 μM (Fig. 8) or 0.5 μM (not shown), or diluent control, was added for 30 minutes to adherent MOs prior to addition of OK-432 for 24 hours. Western blot (Wb) analysis (Fig. 9) shows that Syk kinase becomes phosphorylated following adherence of MOs and is further phosphorylated following OK-432 stimulation but this phosphorylation is substantially reduced in MOs exposed to piceatannol (30 μM) for 30 min before stimulation with OK-432 for an additional 15 min. Addition of piceatannol had no effect on chemokine production, as measured by MCP-1, in human MOs unless it was used at high, unspecific concentrations (120 μM) (Fig. 3B, top). With respect to mRNA, MCP-1 levels are also reduced when exposed for 30 min to 120 μM piceatannol followed by 24-hour incubation with OK-432 (Fig. 3B, bottom). Thus, piceatannol, at high, likely unspecific, concentrations (120 μM) lowers MCP-1 secretion while at low, specific, concentrations (0.5, 1 or 30 μM), has no effect on the tested chemokines, despite exhibiting efficacy in lowering Syk kinase phosphorylation.

The role of PI-3 kinase in OK-432's ability to stimulate MOs was tested where LY294002 (Fig. 8) or Wortmannin (Fig. 3D) was added for 30 min to freshly isolated MOs followed by incubation with OK-432 for 24 hours. Western blot analysis (Fig. 9) shows that PI-3 kinase is not phosphorylated following adherence of MOs but becomes phosphorylated following OK-432 stimulation. This OK-432-dependent PI-3 kinase phosphorylation is decreased when MOs are exposed to LY294002 for 30 min before stimulation with OK-432 for 15 min. Addition of LY294002 (Fig. 8B) or Wortmannin (results not shown) did not significantly reduce MIP-1α, MIP-1β, or MCP-1 production in OK-432-stimulated MOs. Furthermore, a combination of piceatannol (Pic) (30 μM), and LY294002 (LY) (50 μM), or diluent control (ethanol, EtOH), was used throughout the entire MO isolation procedure as well as during incubation with media (Fig. 8C) or OK-432 for an additional 24 hours (Fig. 8D). Incubation with media blocked spontaneous *in vitro *secretion of all tested chemokines, but no significant effect was observed in the ability of MOs to produce MIP-1α, and MIP-1β upon OK-432 stimulation. On the contrary, MCP-1 production appears to be augmented following OK-432 stimulation, pointing to the existence of an additional MCP-1 pathway, dependent on Syk and PI-3 kinase, throughout the isolation and adherence protocol.

### Role of β_2_-integrin (CD18) during stimulation of MOs with OK-432

**Figure 10:**
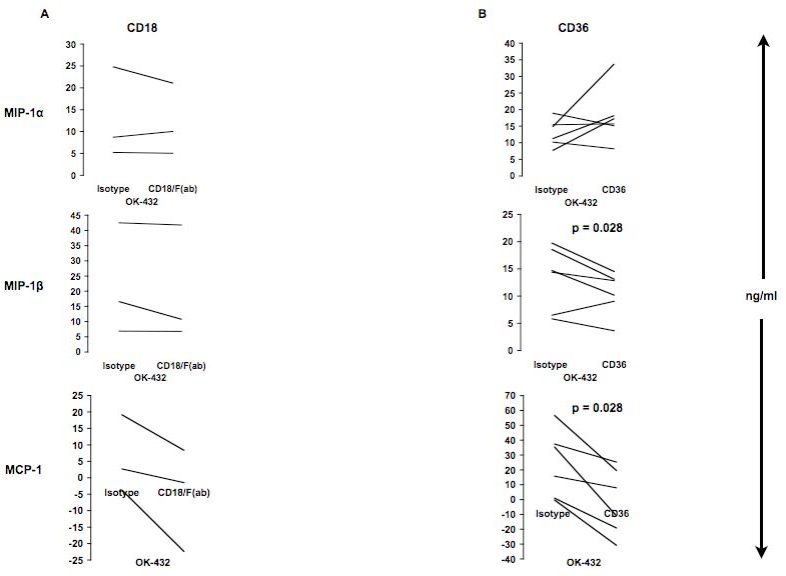
**Role of CD18 and CD36 in OK-432 activation of MOs**. MOs were incubated either with isotype control or anti-CD18/F(ab) (12.5 μg/ml) for 24 h (A). PBMCs were incubated either with isotype control or anti-CD36 (2.5 μg/ml) (B) prior to incubation with OK-432 for 24 h. Supernatants were analyzed for shown chemokines by Multiplex (MIP) or ELISA (MCP-1). A: n = 3; B: n = 6. CD18: MCP-1 (NS).

Treating MOs with a neutralizing F(ab) – anti-CD18 antibody (Fig. 10A) prior to OK-432 stimulation, the secretion of MCP-1 (statistically nonsignificant), but not the other tested chemokines, decreased. Thus, the β_2 _integrin (CD18) may possibly be involved in MCP-1 secretion following OK-432 activation of human isolated MOs.

### Role of CD36 on stimulation of MOs with OK-432

Anti-CD36 antibody or isotype control was added to wells followed by addition of PBMCs, incubated for 40 min, and OK-432 was added for an additional 24 hours (Fig. 10B). CD36 addition decreased MIP-1β and MCP-1 (both p = 0.028) but not MIP-1α secretion. Thus, in MOs, OK-432 elicits both MIP-1β and MCP-1 production in part via CD36 acting as a co-receptor.

## Discussion

We have studied *in vitro *OK-432-stimulated mononuclear MNPs chemokine secretion. Purified MOs, PBMCs or WB secreted MCP-1, MIP-1α, and MIP-1β following OK-432 stimulation at a similar rate, except for a substantially lower MIP-1α secretion from WB. This is in line with other investigators, e.g., showing that *S. pyogenes *directly activates Mϕs to secrete chemokines [24]. Cytokine mRNA quantification of MCP-1 transcripts showed that OK-432 stimulation was followed by cytokine mRNA synthesis within hours. There was a correlation between measured cytokine mRNA levels and cytokine-secreted levels suggesting that OK-432 mainly exerts its effects at the pre-transcriptional level. We furthermore separated by adherence most of the MOs from a sample of PBMCs. Upon OK-432 stimulation both these cell fractions secreted MCP-1 at about twice the level of the MO-depleted fraction. These findings support that MNPs are the main source for MCP-1 within PBMCs. OK-432 also elicited a chemokine response in purified MOs from HNSCC patients.

All studied chemokines were secreted without particular stimulation factors at low adherence conditions, whereas MIP-1α/β secretion was completely inhibited by adherence. At low adherence conditions, the secretion response of MIP-1α/β and MCP-1 from PBMCs and MOs was abolished upon OK-432 stimulation. This might indicate that MNPs undergoing adherence fundamentally alter their chemokine secretion responses and supports that MO to Mϕ differentiation interferes with the regulation of chemokine secretion. This is also supported by our observations of MIP-1α-derived OK-432 stimulated secretion being lower in WB compared to PBMCs and MOs. Diapedesis depends on, e.g., integrin receptor engagement co-triggering differentiation of MOs to Mϕs [18]. Such stimulation may be provided when MOs are isolated by *in vitro *adherence. Syk phosphorylation is a necessary part of β_1–3 _integrin-generated activation [19-21], whereas PI-3 kinase phosphorylation is required in β_2 _integrin-generated activation [20]. We have presently shown that adherence of MOs stimulated phosphorylation of Syk but not PI-3 kinase, as determined by Western blots. Thus, it appears that possibly both β_1 _and α_v_β_3 _integrins, but not β_2 _integrin, play a role in mediating MO adhesion. Based on Western blots, binding of OK-432 to MOs elicited a higher Syk phosphorylation compared to unstimulated MOs, possibly suggesting an adherence-mediated priming effect. PI-3 kinase phosphorylation occurred only upon stimulation with OK-432. We have shown that addition of either one of these inhibitors to OK-432-stimulated MOs did not change chemokine secretion. On the contrary, with addition of both of these inhibitors, MCP-1 secretion was increased, pointing to the existence of other possible signaling pathways. In addition, presence of both inhibitors throughout the isolation and separation of monocytes blocked the unstimulated secretion of chemokines supporting that β integrins are important for chemokine secretion during the adhesion process. Our observed Syk phosphorylation upon OK-432 binding pointed towards an integrin dependence and resonated with Cuzzola et al [22], who have shown that the MO interleukin response to Group A Streptococci (GAS) was dependent on the β_2 _integrin. We explored whether this applied to the OK-432-mediated activation of MOs by adding a blocking anti-CD18 antibody. MCP-1 production appears to be partially inhibited by the anti-CD18 antibody. Thus, the β_2 _integrin may play a role in mediating the effects of OK-432 on MOs. Nevertheless, other receptors may phosphorylate either Syk or PI-3 kinase or both and their nature has yet to be established.

OK-432 probably stimulates MOs via TLR2 [15] and/or TLR4 [25]. In order to study the role of these receptors, MOs were stimulated in parallel with OK-432, and the known TLR2 or TLR4 agonists, LTA and LPS, respectively. Results showed that MOs respond to OK-432 in a similar fashion to LTA stimulation, thus, arguing for primary TLR2-dependent stimulation of MOs by OK-432.

A potential candidate for co-stimulation of MOs is CD36. This receptor has been found to function as a co-receptor for TLR2 receptor [26]. We determined a reduced MIP-1β and MCP-1, but an unchanged MIP-1α response upon employing blocking anti-CD36 antibodies. Thus, CD36 may function as an important receptor in the OK-432 stimulation of MOs with respect to chemokine secretion.

Other co-stimulating receptors than the presently shown ones may be important for chemokine production following OK-432 stimulation of MNPs. Examples include CD62L (L-selectin receptor), a mediator of MOs rolling on and interacting with endothelial cells [27] and CD162 (P-selectin glycoprotein ligand-1), a promoter in the interaction between MOs and platelets [28]. Future studies are needed to establish their roles in OK-432 stimulation in MOs.

The shown MNP stage-dependence for MIP-1α secretion and adherence-dependent chemokine response to OK-432 supports the hypothesis that chemokine secretion is less favoured when MOs are stimulated by OK-432 in pb compared to extravasal stimulation. This is in line with our previous observation that TNF-α is primarily secreted upon OK-432 stimulation after initiation of MO differentiation [17]. The present report thus adds more support to the notion that OK-432 is a target-seeking substance. Only when MNPs are stimulated by, e.g., tumor cells or intercellular substances [29], one observes a chemokine response. The present results argue in favor of chemokine secretion being an important effector function of MNPs during OK-432 stimulation. This is important as to designing future BRM treatments with OK-432. Platinum-based drugs, e.g., negatively interfere more with monocyte than lymphocyte function [31] and should thus be avoided in combination with OK-432 treatment.

MIP-1α/β and MCP-1 secretion is induced by stimulation of MOs with OK-432. These chemokines are ligands of the receptors CXCR1, CCR1, CCR2 and CCR5, present on polymorphonuclear granulocytes, basophiles, granulocytes, MOs, Mϕs, DCs, NK cells, T, and B cells [32]. Thus, the shown chemokine secretion after OK-432 stimulation of MOs may co-explain the systemic effects of OK-432 on the immune system. Similar findings were made by Veckman et al [24] upon stimulating Mϕs with pathogenic live bacteria (serotype T1M1 (IH32030)) isolated from a child with bacteremia. In contrast, we have stimulated monocytes with a heat- and penicillin G-killed, low-virulence SU strain of *S. pyogenes *and found very low levels of RANTES (CCL5). Therefore, it is plausible that virulence status/degree, strain, and preparation of bacteria influence chemokine production and MOs may have a different chemokine profile compared to Mϕs. This becomes especially interesting when the emphasis is on OK-432 being a lyophilized, killed *S. pyogenes*. *S. pyogenes *is an important pathogen causing human disease. The diseases caused by *S. pyogenes *range from tonsillitis, impetigo, and scarlet fever to lethal toxic shock syndrome [13]. The shown mechanisms of MNP activation by OK-432 thus shed light on these diseases as well.

## Conclusion

We have found that MCP-1 and MIP-1α/β production by OK-432-stimulated human purified adherent MOs occurs in most healthy individuals. MIP-1α secretion is mostly found following differentiation stimuli of MOs to become Mϕs. Furthermore, MOs stimulated with OK-432 respond with chemokine secretion dependent on adherent growth conditions. OK-432 may rely on β_2 _integrin stimulation. CD36 modulates the MIP-1β and MCP-1 response to OK-432. To some extent OK-432 may act as a target-seeking substance whereby only MOs adhered, e.g., to a target, secrete substantial amounts of chemokines in part explaining why OK-432 is suited as a BRM drug.

## Methods

### Reagents and antibodies

OK-432 (Picibanil) (Chugai Pharmaceutical Co. Ltd., Tokyo, Japan) was dissolved in sterile water as a stock solution with a concentration of 1 KE (Klinische Einheit)/ml = 0.1 mg/ml. LTA from *S. pyogenes *and LPS from *E.coli *026:B6 (Sigma, St.Louis, MO, USA) were dissolved in PBS as a stock solution of 5 mg/ml (LTA) and 25 μg/ml (LPS) and used at a final concentration of 0.5 μg/ml and 1 μg/ml, respectively. The Syk inhibitor piceatannol (Calbiochem, La Jolla, CA, USA) was dissolved in ethanol as a stock solution with a concentration of 2 mg/ml (8000 μM) and used at a final concentration of 0.5, 1, 30 or 120 μM [18,19,33]. The PI-3 kinase inhibitor, LY294002 (Sigma) was dissolved in sterile water at a concentration of 5 mg/ml (14.5 mM) and used at a final concentration of 50 μM. The PI-3 kinase inhibitor, wortmannin (Sigma) was dissolved in DMSO at a concentration of 1 mg/ml (2.3 mM) and used at a final concentration of 100 nM. Azide-free, blocking mouse anti-human CD36 (HyCult, Uden, The Netherlands) came as a 100 μg/ml stock solution and anti-mouse rat IgG_1 _isotype control (R&D Systems Europe Ltd., Abingdon, Great Britain) was dissolved in sterile PBS as a 250 μg/ml stock solution. F(ab) (degraded by pepsin (Sigma)), azide-free, blocking rat anti-human CD18 (Chemicon International, Temecula, CA, USA) was dissolved in sterile PBS as a 100 μg/ml stock solution. Anti-rat IgG_2B _isotype control (R&D Systems Europe Ltd.) was dissolved in sterile PBS as a 500 μg/ml stock solution.

### Patients and controls

Patients admitted to surgery for head and neck squamous cell carcinoma (HNSCC) gave written consent before participating in the study. All patients were males. Patient 1 was 60 years and had tonsillar carcinoma (T_3_N_0_M_0_G_1_). Patient 2 had carcinoma of the tongue (T_2_N_2_M_0_G_2_). Patient 3 had carcinoma of the basal tongue (T_2_N_2_M_0_G_2_). Patient 4 had laryngeal carcinoma (T_4_N_1_M_0_). All patients donated blood approximately 2 weeks post-operatively as is appropriate in the event of starting treatment with OK-432. Whole blood from controls was obtained from randomly selected, healthy donors at Haukeland University Hospital. As selection was done randomly, age and sex of donors were not taken into consideration, i.e. not recorded. The study was approved by the Regional Committee for Medical Ethics.

### Whole blood, PBMC, and monocyte preparation

Whole blood was diluted 1:1 with Ultraculture (Lonza, Basel, Switzerland), allocated to a 24-well plate (Nunc A/S, Roskilde, Denmark) and upon experiment completion, the supernatant was collected after the whole blood/Ultraculture mixture was centrifuged. PBMCs were separated by density gradient centrifugation with Lymphoprep^® ^(Nycomed, Oslo, Norway) as density gradient medium in LeukoSep tubes (Greiner-Bio One GmbH, Frickenhausen, Germany). MOs were, unless otherwise stated, isolated from blood by gradient centrifugation followed by adherence to plastic. The PBMC yield of 7 ml blood (6 × 10^5 ^cells/well) was allocated to a 24-well plate (Nunc) with RPMI 1640 (Lonza) supplemented with amphotericin B (2.5 μg/ml) and glucose (2% v/v) (both Sigma), HEPES (1% v/v), L-glutamine (1% v/v), penicillin (100 IU/ml), streptomycin (100 μg/ml), sodium bicarbonate (2.7% v/v), sodium pyruvate (1% v/v)(all from Lonza) and 20% AB serum to a total volume of 0.5 ml/well. After 40 minutes pre-incubation, the adherent MOs were purified by washing, and then cultured in complete RPMI 1640 (Lonza)/20% AB serum with 0.5 ml/well. The method yields in general cultures which contain at least 75% MOs, as determined by nonspecific esterase stain (NSES), and more than 90% viable cells, as tested by tryphan blue stain. In some experiments, MOs were also isolated using the negative isolation kit (Dynal Biotech, Oslo, Norway) according to the manufacturer's protocol. Briefly, PBMCs, isolated by gradient centrifugation using Lymphoprep-containing LeukoSep tubes, were resuspended in PBS/0.1% bovine serum albumin (BSA) (Sigma) and exposed to an equal mixture of blocking reagent and antibody mix for 10 min at 4°C. After 10 min, PBMCs were washed, added fresh PBS/0.1% BSA, added to washed beads, and incubated for 15 min with gentle tilting and rotation at 4°C. After incubation, resuspended rosettes were pipetted and placed in the Dynal MPC magnet for 5 min. The supernatant was collected and placed in the Dynal MPC magnet for an additional 2 min. The collected MO-pure supernatant (95% purity – NSES) was washed, cells counted, and added to a 24-well plate.

### Experimental conditions

In different experimental series, MOs (1 × 10^5 ^cells/well) were cultured either with piceatannol (0.5, 1, 30 or 120 μM – final concentration), LY294002 (50 μM – final concentration), wortmannin (100 nM – final concentration) or anti-CD36 (2.5 μg/ml – final concentration) or anti-mouse IgG_1 _isotype control (2.5 μg/ml – final concentration) or pepsin-degraded (Fab) anti-CD18 (12.5 μg/ml – final concentration) or anti-rat IgG_2B _isotype control (12.5 μg/ml – final concentration) for 30 min before OK-432 (0.01 KE/ml – final concentration) was added and after 24 hours the supernatants were collected and stored at -80°C for further analysis.

### Total RNA extraction

In some experiments, total RNA was isolated immediately after harvesting MOs (24 hours post-stimulation), using the RNeasy Minikit's (Qiagen, Hilden, Germany) protocol for isolation of total RNA from animal cells grown in monolayer, as recommended by the manufacturer. Briefly, adherent MOs were added 350 μl lysis buffer (buffer RLT containing beta-mercaptoethanol (Sigma)), homogenized by vortexing for 30 s, and the homogenate was applied to an Rneasy spin column after addition of ethanol in order to bind total RNA to the membrane. Contaminants were removed by washing with buffers RW1 and RPE, and total RNA was eluted using water. In addition, total RNA was isolated from MOs of normal blood donors and subsequently pooled for use in generating the standard curve.

### Reverse transcription

TaqMan RT reagents (Applied Biosystems, Foster City, CA, USA) were used to reverse transcribe total RNA from OK-432-stimulated MOs obtained from normal blood donors. Final concentration of the RT reaction was 1 × TaqMan RT buffer, 5.5 mM MgCl 2, 2 mM dNTP mixture (500 μM of each dNTP), 2.5 μM random hexamers, 0.4 units/μl RNase inhibitor, 2.5 units/μl Multiscribe RT, 5–25 ng RNA, and DEPC water to a total volume of 50 μl. The RT thermal cycling was performed on a Mastercycler gradient thermal cycler (Eppendorf, Hamburg, Germany) under the following conditions: primer incubation for 10 min at 25°C, reverse transcription for 30 minutes at 48°C, and inactivation of reverse transcriptase for 5 minutes at 95°C.

### Primer and probe design

Primer and probes for the TaqMan assays were designed using the Primer Express software (Applied Biosystems). Intron-spanning primers were used to prevent amplification of genomic DNA. The candidate genes were MCP-1 [GenBank: NM_002982] (70 bp-amplicon); primers: (F) 5'-CTCTCGCCTCCAGCATGAA-3', (R) 5'-GGAATGAAGGTGGCTGCTATGA-3', probe: 5'-CCGCCCTTCTGTGCCTGCTGC-3'and the housekeeping gene used was CD68 [GeneBank: BC015557] (67 bp-amplicon); primers: (F) 5'-CCCCACGCAGCAAAGTG-3', (R) 5'-CCAGGGGTGCTTGGAGATCT-3, probe: 5'-TCTCGGCTCAGAATGCATCCCTTCG-3'). Designed primers and probes were purchased from MedProbe (Oslo, Norway).

### Real-time quantitative RT-PCR analysis

The ABI PRISM 7700 Sequence Detection System (Applied Biosystems) with 96-well plates was used to perform real-time quantitative RT-PCR. Reactions were carried out in a total volume of 25 μl containing 12.5 μl of 2 × TaqMan Universal PCR Master Mix (Applied Biosystems), 300 nM (0.25 μl) forward primer, 300 nM (0.25 μl) reverse primer, 200 nM (1 μl) TaqMan probe, 8 μl water, and 3 μl cDNA template. Thermocycler conditions were as follows: incubation for 2 minutes at 50°C, followed by incubation for 10 minutes at 95°C and 40 cycles of two-step (denaturing for 15 seconds at 95°C followed by annealing/extension for 1 minute at 60°C) PCR. As a reference gene, CD68 was used to normalize the gene expression of MCP-1. To generate a standard curve for relative gene expression determination, cDNA synthesized from pooled total RNA of MOs obtained from healthy blood donors was used. The standard curves generated for the cDNA analyzed had slopes with a mean of -3.3.

### Western blot analysis

MOs from 7 ml blood were isolated by gradient centrifugation in Lymphoprep-containing LeukoSep tubes, followed by the monocyte negative isolation kit (Dynal Biotech), allowed to adhere for 40 minutes, after which piceatannol or LY294002 or diluent control was added for 30 min, followed by incubation with OK-432 for 15 min. Upon experiment completion, cells were washed once with cold PBS, added lysis buffer (50 mM Tris-HCl, pH 7.4, 1% NP40, 0.25% sodium deoxycholate, 150 mM NaCl, 1 mM EDTA, 1 mM PMSF, 1 mM sodium orthovanadate, 1 mM NaF, and 1× complete proteinase inhibitor (Roche, Nutley, NJ, USA)) and scraped gently using a cell scraper. The cell lysate was clarified by centrifugation at 16,000 g for one minute at 4°C. Protein levels were measured using the Bio-Rad BCA Protein Assay (Bio-Rad, Hercules, CA, USA). Samples were added 1× loading buffer and the lysate was boiled for 5 minutes. Samples (6 μg/lane) and a Precision Plus Protein Kaleidoscope standard (Bio-Rad) were electrophoresed on a 10% SDS polyacrylamide gel for 60 minutes at 200 V and transferred to nitrocellulose membranes (Bio-Rad) in electroblotting buffer (25 mM Tris base, 0.2 M glycine, and 20% methanol) for 60 minutes at 100 V. Membrane was blocked with 5% BSA in TBS-Tween for one hour at RT with gentle tilting. The blots were exposed either to a polyclonal antibody against phosphorylated Syk or PI3 kinase p85/p55 or total Syk or total PI3 kinase p85 (all: Cell Signaling, Beverly, MA, USA) (all: 1:1000 in 5% BSA in TBS-Tween) overnight at 4°C with gentle shaking. After washing, an HRP-linked goat anti-rabbit IgG (H + L) antibody (Bio-Rad) (1:2000) was added for one hour at room temperature with gentle tilting. Proteins were detected using luminol/enhancer/hydrogen peroxide – based ECL and visualized on the Molecular Imager ChemiDoc XRS System (Bio-Rad).

### Multiplex cytokine analysis

Chemokines in supernatants were detected using the Luminex immunobead technology. A 25-plex or 5-plex chemokine kit (all from Invitrogen/Biosource, Carlsbad, CA, USA), were used to analyze cytokine levels in supernatants from successfully completed experiments. In short, antibody-coupled beads were incubated with target analyte after which they were incubated with biotinylated detection antibody before finally being incubated with streptavidin-phycoerythrin. Standard sensitivity was 10 pg/ml (MIP-1α, MIP-1β). Samples were then read by the Bio-Plex array reader (Invitrogen/Biosource), using Luminex fluorescent bead-based technology (Luminex Corporation Austin, TX, USA).

### MCP-1 protein analysis

The contents of MCP-1 in supernatants were determined by enzyme-linked immunosorbant assay kit (ELISA) manufactured by R&D Systems (R&D Systems). All procedures were performed according to the specifications of the manufacturer. In short, 96-well microtiter plates (Costar Corning, NY, USA) were coated overnight at room temperature with monoclonal mouse anti-human MCP-1 capture antibodies. Diluted samples and recombinant human MCP-1 standard were added and incubated for 2 h at room temperature followed by addition of biotinylated polyclonal goat anti-human MCP-1. The plates were incubated for 20 minutes at room temperature with streptavidin-conjugated horseradish peroxidase. Tetra-methyl-benzidine (TMB) (Sigma) and H_2_O_2 _were used as substrate. Absorbency values were measured at 450 nm using Softmax Pro version 4.0 on an Emax Precision microtiter plate reader (Molecular Devices, Sunnyvale, CA, USA). The lower detection level was 15.62 pg/ml. Chemokine levels obtained with media controls (after 24 h) were substracted from those obtained with OK-432-, LTA-, LPS-stimulation. All media controls tested directly after monocyte purification had nondetectable chemokine levels.

### Statistical analyses

The statistical program package Statistical Package for the Social Sciences (Ver. 16 SPSS, Chicago, Ill., USA) was used. Chemokine levels and expression were compared by the Wilcoxon signed-rank test (two-tailed). Differences were considered significant at *p *< 0.05.

## Abbreviations

BRM: biological response modifier; HNSCC: head and neck squamous cell carcinoma; MIP: macrophage inflammatory protein; MCP: monocyte chemoattractant protein; pb: peripheral blood; PI: phosphoinositide; pSyk: phosphoSyk; TLR: Toll-like receptor.

## Competing interests

The authors declare that they have no competing interests.

## Authors' contributions

CO performed the Western blot analysis, Multiplex analysis, all the experiments and data analysis. HS did the RT-PCR experiments and analysis. KB operated the Luminex machine. JO provided OK-432. CO and HJA were responsible for the concept and design of the experiments and primarily writing the manuscript. All authors have read and approved the manuscript.
